# 

**Published:** 1906-09-08

**Authors:** 


### THE HOSPITAL

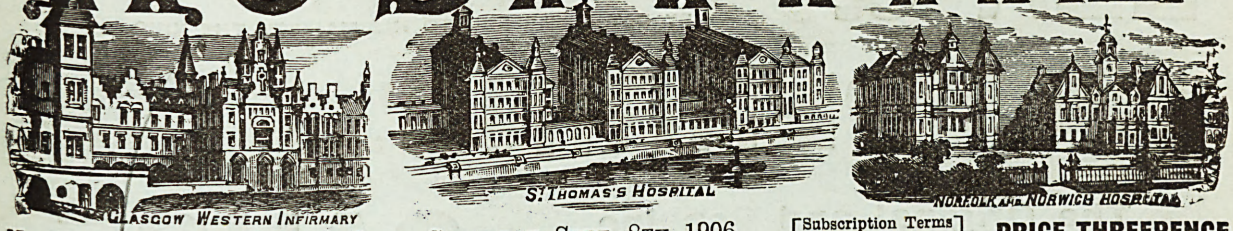

No. 1,042. Vol. XL. [Registered as a Newspaper.] SATURDAY, SEPT. 8TH, 1906. [Subscription Terms see p. 414.] PRICE THREEPENCE

